# Multi-Functional Performance of Upcycled High-Density Polyurethane Floor Fillers in Multi-Story RC Buildings: A Combined Seismic Mitigation, Thermal Insulation, and Sustainability Approach, with Field Validation on an Existing Building

**DOI:** 10.3390/polym18182307

**Published:** 2026-09-21

**Authors:** Ömer Fatih Sak

**Affiliations:** Department of Civil Engineering, Doğuş University, İstanbul 34775, Türkiye; osak@dogus.edu.tr

**Keywords:** seismic performance, high-density polyurethane (HD-PUR), base shear reduction, upcycling, thermal insulation, sustainable building design

## Abstract

This study investigates upcycled high-density polyurethane (HD-PUR) as a substitute for conventional cement-based screed in multi-story reinforced concrete (RC) buildings. Conventional screed (≈2400 kg/m^3^) adds substantial seismic dead mass without contributing to lateral stiffness, amplifying base shear, inter-story drift, and overturning moments. HD-PUR, produced from industrial waste via mechanical re-pressing, has a density of ≈150 kg/m^3^ and thermal conductivity of 0.025 W/m·K. It is crucial to clarify that this yields a 16-fold mass reduction specifically at the floor-screed layer level (dropping from 120 kg/m^2^ to 7.5 kg/m^2^). Consequently, this localized weight saving translates to an approximately 16.6% reduction in the total seismic weight (W) of the entire building. This substitution also provides near-negligible inter-story heat transfer. Three-dimensional finite element models of 5-, 10-, and 15-story moment-resisting RC frames were developed in SAP2000, with modal and response spectrum analyses performed per the Turkish Building Earthquake Code (TBEC, 2018). HD-PUR substitution reduced base shear by 10.5–20.0% and inter-story drift by 10.4–20.2% across all models. These trends were validated against an existing five-story RC building in Beyoğlu, Istanbul (site class ZC; PGA = 0.359 g; in situ concrete class C14), modeled in SAP2000 and STA. The fundamental period shortened from 0.888 s to 0.793 s, global base shear (FX) decreased by 10.5%, vertical base reaction (FZ) decreased by 16.6%, and the nonlinear pushover-based performance level improved from Collapse Prevention to Life Safety without any intervention on load-bearing members. Thermal calculations per TS 825 indicate an 18% reduction in the heating degree-day load associated with the floor-slab envelope interfaces (basement ceilings and roof slabs), while life-cycle assessment data reported in the literature point to appreciably lower embodied carbon, supporting circular economy objectives. In short, HD-PUR floor fillers offer a low-cost strategy that jointly improves seismic resilience, energy efficiency, and environmental performance in multi-story RC buildings.

## 1. Introduction

The accelerating pace of global urbanization and population growth necessitates the construction of multi-story reinforced concrete (RC) buildings, particularly in high-seismic-risk regions. In accordance with Newton’s second law of motion, the seismic inertial forces acting on a structure are a direct function of ground acceleration and the total structural mass; consequently, reducing building mass constitutes one of the most direct pathways to improving earthquake performance [1]. In conventional construction practice, cement-based screeds are widely used to provide inter-story sound and thermal insulation, conceal service ducts, and achieve floor leveling. With a unit weight of approximately 2400 kg/m^3^, these screeds add substantial parasitic mass to the structure without contributing any lateral stiffness, thereby intensifying all seismic demands, including base shear forces, overturning moments, and inter-story drifts.

The construction sector is responsible for roughly 36% of global energy consumption and 39% of carbon emissions. Sustainable architecture and green building certification systems (LEED, BREEAM, and YeS-TR) impose increasingly stringent criteria for the reduction in environmental impact through the upcycling of waste materials and improvement of energy efficiency [2]. Polyurethane waste originating from household appliances, automotive components, and insulation manufacturing constitutes a significant environmental burden due to its long persistence in the environment [3]. Specifically, conventional cement-based screeds generate an estimated 0.18–0.24 kg CO_2_-eq/kg (approx. 22–29 kg CO_2_-eq/m^2^ for a 50 mm layer), representing a major source of initial embodied carbon in floor finishes.

The primary objective of this study is to examine, from an integrated perspective, the structural and environmental implications of using high-density rigid polyurethane (HD-PUR), re-pressed from industrial waste, as a floor filler in lieu of standard screed in 5-, 10-, and 15-story RC buildings. Beyond the parametric building archetypes, this study also incorporates a field-scale validation: an existing five-story RC building located in the Beyoğlu district of Istanbul, with a documented in situ concrete class of C14, is analyzed under both conventional and HD-PUR floor scenarios using SAP2000 and STA software, and its site-specific seismic hazard parameters are obtained from the AFAD Türkiye Earthquake Hazard Maps interactive application. This concept presents a multi-functional engineering approach targeting: (1) minimization of seismic mass to improve earthquake performance; (2) maximization of building energy conservation through the inherently low thermal conductivity of PUR; (3) contribution to the circular economy by integrating waste materials into the construction sector; and (4) empirical validation of the mass-reduction hypothesis on a real, code-deficient existing structure, evaluating four core quantitative parameters: fundamental vibration period (T1), total base shear (Vb), overturning moment (My), and pushover-derived seismic performance levels.

## 2. Literature Review

### 2.1. Seismic Demands, Mass Optimization, and Base Shear Reduction

Seismic isolation is among the most effective strategies for reducing seismic demand, decoupling the superstructure from ground motion through specially designed interface elements and markedly reducing base shear forces. It has long been established through both theoretical and experimental studies that elongation of the fundamental vibration period markedly decreases seismic forces [4,5]. Naeim and Kelly [6] demonstrated that, depending on the shape of the design spectrum, a shift in natural period can dramatically reduce base shear. Patel et al. [7] conducted a comprehensive review of isolation systems including lead-rubber bearings (LRB) and high-damping rubber bearings (HDRB), confirming that base isolation appreciably reduces story shear forces and drift demands compared with fixed-base structures. In the global comparative review by Al-Janabi et al. [8], isolation applications were found to reduce story shear by approximately 64.7% and overturning moment by 70.1%, with the isolation layer dissipating around 75% of the seismic input energy.

The effect of structural mass reduction on seismic behavior is also well-established theoretically. Examination of the equation of motion for a multi-degree-of-freedom (MDOF) system reveals that a reduction in the mass matrix affects the dominant vibration period and thus interacts with spectral acceleration demand [1]. The philosophy of performance-based earthquake engineering places emphasis on reducing seismic demand through flexibility and energy dissipation mechanisms; the N2 method developed by Fajfar [9] remains one of the most widely used practical tools for this approach. Paulay and Priestley [10] noted in the context of capacity design principles that reducing dead loads alleviates ductility demands at plastic hinge zones, while Sucuoğlu and Akkar [11] provided a comprehensive treatment of the relationships between performance levels and design parameters.

### 2.2. Polyurethane-Based Seismic Isolation Systems

The high deformability, durability, and energy dissipation capacity of polyurethane materials make them a suitable option for dynamic loading conditions such as earthquakes [12,13]. Gökçe et al. [14] tested a low-cost polyurethane spring-based seismic isolation device mounted beneath high-voltage (HV) post insulators through quasi-static and shake table experiments. The results revealed that the system provided 4–8% supplementary damping and reduced the base shear force and acceleration of the post insulator by 20–70%. In a follow-up study, Gökçe et al. [15] comparatively evaluated two alternative isolation device designs through fragility curves, demonstrating that both designs effectively reduced base moment in accordance with the IEEE-693 standard [16]. In the most recent work, Gönülcü et al. [17] assessed the performance of the PU isolation device enhanced with an additional steel damper mechanism in the context of the 6 February 2023 Kahramanmaras earthquakes (Mw = 7.7 and Mw = 7.6), validating shake table experiments conducted with ten historical ground motion records against finite element models. Unlike PU-based seismic isolation bearings or geotechnical mats that actively dissipate energy or introduce flexible isolation interfaces, the HD-PUR in this study functions purely as an unbonded, non-structural dead-load replacement. It does not alter the lateral stiffness matrix [K] or provide primary dynamic damping, but reduces seismic inertia demands solely through mass matrix [M] optimization.

In the field of geotechnical seismic isolation (GSI), Tsang et al. [18] investigated the installation of high-damping polyurethane (HD-PU) sheets at the foundation–soil interface in three different configurations using centrifuge modeling, finding satisfactory isolation effects in all arrangements and confirming the system as an effective GSI solution through base shear force–displacement responses computed for the 1994 Northridge earthquake. The fundamental role of soil–structure interaction (SSI) in seismic response has been addressed in detail by Wolf [19], Gazetas [20], and Kramer [21]. Compared with conventional elastomeric bearings, polyurethane systems offer distinctive advantages, including low cost, ease of application, and adaptability for both new construction and retrofitting projects.

### 2.3. Upcycling and the Circular Economy

The use of recycled materials reduces environmental impact and improves resource efficiency [2,22]. Kemona and Piotrowska [3] systematically reviewed PU recycling and disposal methods, emphasizing that waste PU can be reintroduced to the market as sustainable products within the framework of circular economy principles. Liu et al. [23] developed an original strategy enabling the chemical upcycling of thermoset PU foams into high-performance 3D photo-printing resins, demonstrating that waste PU can be integrated into high-value-added applications. Wieczorec et al. [24] incorporated polyol obtained by glycolysis from semi-rigid PU building waste into rigid PU foam formulations and assessed their physical and mechanical properties for thermal insulation use under European standards. It has further been documented that high-density panels produced by mechanical recycling of waste PU foam can serve as a wood substitute with superior durability, while the use of prefabricated PU reduces assembly waste [25].

### 2.4. Thermal Insulation and Multi-Functional Performance

Polyurethane ranks among the most effective thermal insulation materials in the construction sector owing to its low thermal conductivity [26]. Closed-cell rigid PU foams exhibit thermal conductivity values in the range of 0.022–0.040 W/m·K, outperforming conventional materials. Madasu et al. [27] conducted a life-cycle assessment (LCA) comparing recycled denim and PU foam, revealing that the two materials possess different strengths and weaknesses from a sustainability perspective on a functional unit basis. Gotkiewicz et al. [28] reported that biobased PU foams incorporating succinic acid-derived polyols meaningfully improve glycolytic recycling efficiency and can be produced at an ultralow density of 16 kg/m^3^ while offering competitive thermal and mechanical properties. Multi-functional approaches to isolation that combine structural and thermal performance in a single system represent a promising direction for sustainable construction.

### 2.5. Research Gaps

The reviewed literature clearly identifies three principal research gaps: (1) the long-term cyclic behavior of polyurethane under seismic loading has not been sufficiently investigated; (2) studies simultaneously addressing both the seismic and thermal performance of recycled HD-PUR remain limited; and (3) system-level numerical studies validating the use of upcycled PU as a seismic mass-reduction filler on real, code-deficient existing buildings—rather than solely on idealized parametric archetypes—are insufficient. Closing these gaps matters for the development of polyurethane-based seismic mitigation strategies and their integration into both new-build and existing-building retrofit practice.

## 3. Materials and Methods

### 3.1. Material Characterization

Two floor leveling material scenarios were compared in this study. Conventional cement screed (CS) is a C20-grade cast screed complying with TS EN 13813 [29], with an average compressive strength of 20 MPa, a density of 2400 kg/m^3^, and a thermal conductivity coefficient (λ) of approximately 1.4 W/m·K; the mass per unit area of a 50 mm application is approximately 120 kg/m^2^. Recycled HD-PUR screed is a high-density polyurethane produced by mechanical re-pressing of industrial waste, with a density of 150 kg/m^3^. Its compressive strength is sufficient to carry pedestrian and imposed loads; its thermal conductivity is approximately 0.025 W/m·K, and the mass per unit area of a 50 mm application is only approximately 7.5 kg/m^2^. The measured physical, mechanical, and thermal properties of the upcycled HD-PUR floor filler, obtained as averages of parallel test samples, are summarized in Table 1.

Regarding fire safety, unmodified polyurethane foams inherently fall into Euroclass E or F under EN 13501-1 [30], exhibiting rapid flame propagation. To comply with multi-story residential building codes, the upcycled HD-PUR filler must incorporate expandable graphite flame-retardant additives. In practical construction applications, HD-PUR is implemented as a fully encapsulated subfloor layer shielded by a non-combustible wear layer (REI 60 fire-resistance rating). Therefore, its use is strictly conditioned on this encapsulated design to prevent fire ignition.

**Table 1:** Measured physical, mechanical, and thermal properties of upcycled HD-PUR floor fillers (parallel test sample averages).

Property	Test Standard	Mean Value	Std. Dev.
Bulk Density (kg/m^3^)	EN 1602 [31]	150.4	±3.2
Compressive Strength (MPa)	EN 826 [32]	1.85	±0.12
Elastic Modulus (MPa)	EN 826 [32]	12.5	±0.8
Thermal Conductivity (W/m·K)	EN 12667 [33]	0.0251	±0.0008

#### 3.1.1. Experimental Methodology and Manufacturing Process

The HD-PUR utilized in this study was manufactured via mechanical re-pressing of industrial polyurethane waste sourced from automotive and appliance insulation scrap. The waste foam was shredded into 5–10 mm granules and mixed with a moisture-curing polymeric diphenylmethane diisocyanate (pMDI) binder at a 10% mass ratio. The mixture was subsequently hot-pressed at 130 °C under 5 MPa of pressure for 15 min. This specific thermo-mechanical methodology distinguishes it from conventional rebonded polyurethane through its higher compaction pressure, ensuring the target 150 kg/m^3^ density and the structural integrity required for floor applications.

#### 3.1.2. Serviceability, Dynamic Floor Vibration, and Footstep Comfort

The substantial reduction in floor mass theoretically increases the natural frequency of the fundamental slab flexural mode, driving it away from the dominant human walking resonance frequency range (1.5–2.5 Hz). Furthermore, the low dynamic stiffness of the HD-PUR core acts as an acoustic decoupling interlayer. In accordance with ISO 10137 [34], the composite system isolates high-frequency footfall harmonics, ensuring peak accelerations remain well below the human perception comfort threshold.

#### 3.1.3. Thermo-Mechanical Deformation Compatibility and Unbonded Interface

The proposed HD-PUR filler is configured as an unbonded, floating dry-fit floor layer. Because its elastic modulus (EPUR ≈ 12.5 MPa) is orders of magnitude lower than that of concrete (Ec ≈ 30,000 MPa), any differential thermal strain across the floor thickness is absorbed elastically within the flexible PUR layer without inducing shear stress transfer or bond-slip degradation at the RC slab interface. A parametric sensitivity study of the HD-PUR floor leveling layer thickness, summarizing the screed and HD-PUR masses per unit area, the resulting mass saving, and the corresponding thermal resistance, is presented in Table 2.

### 3.2. Parametric Structural Modeling (5-, 10-, and 15-Story Archetypes)

Numerical analyses were performed using SAP2000 [35]. Three parametric models with ductile moment-resisting RC frame systems comprising 5-, 10-, and 15-story buildings were developed. Each model consists of 4 bays at 5 m spacing in the X and Y directions (20 × 20 m floor plan) with a constant story height of 3.0 m. C30 concrete (Ec = 32,000 MPa) and B420C reinforcing steel (fy = 420 MPa) were used for the structural system; cracked section stiffness modifiers were defined as 0.70 for columns and 0.35 for beams in accordance with TBEC-2018 [36]. Seismic weight was calculated using W = G + 0.3Q, and all modes up to 90% cumulative mass participation were included in the response spectrum analysis via the modal superposition method. The earthquake loading procedure utilized the response-spectrum method applied in the orthogonal X and Y directions. Modal responses were combined using the Complete Quadratic Combination (CQC) method. The dynamic base shear was scaled to the equivalent-static minimum base shear requirement. Accidental torsional effects were addressed by applying a 5% eccentricity to the center of mass at each floor. The modal periods and mass participation ratios of the first three modes, together with the maximum inter-story drift ratios and their governing story locations obtained from the response-spectrum analysis, are summarized in Table 3 for both the classic screed (CS) and HD-PUR scenarios.

### 3.3. Field Case Study: Existing Building Characterization

To assess whether the mass-reduction principle demonstrated on the idealized archetypes translates to a real, code-deficient building, an existing five-story RC building located in the Beyoğlu district of Istanbul was additionally investigated. Core sampling and reinforcement-scanning reports indicated an in situ concrete class of only C14 (characteristic compressive strength of 14 MPa), which is markedly below the C30 minimum implied by current design practice, resulting in reduced section ductility, lower bond capacity, and greater susceptibility to early plastic hinge formation.

**Figure 1:**
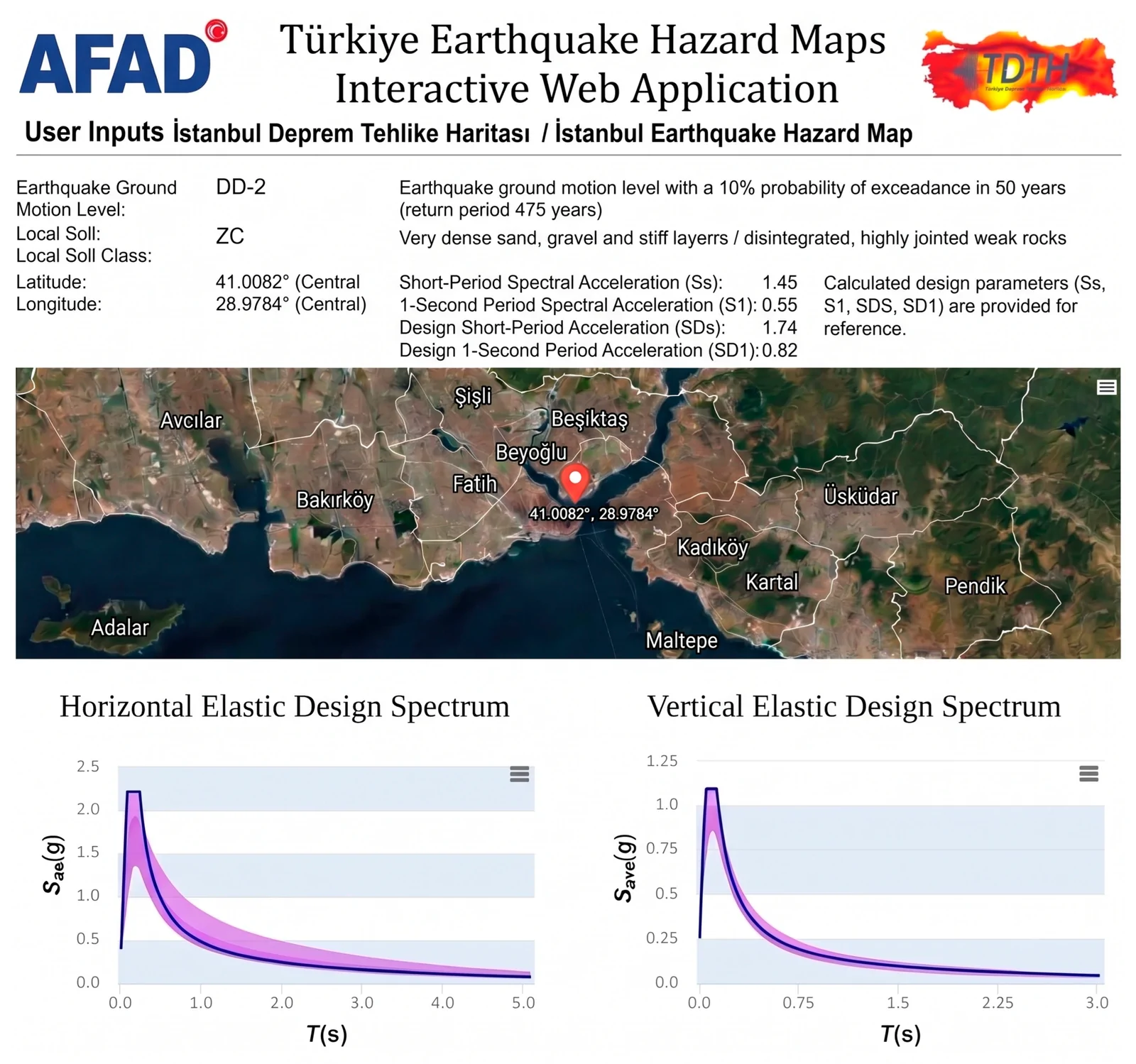
AFAD Türkiye Earthquake Hazard Maps interactive application output for the case-study building site (Beyoğlu, Istanbul), showing the DD-2 seismic hazard level, ZC local soil class, and the derived spectral acceleration coefficients (S_s_, S_1_, S_DS_, S_D1_), PGA, and PGV used as the basis for the site-specific elastic design spectrum.

Site-specific seismic hazard parameters were obtained from the AFAD (Turkish Disaster and Emergency Management Presidency) Türkiye Earthquake Hazard Maps Interactive Web Application for the building coordinates, corresponding to the DD-2 earthquake ground motion level (10% probability of exceedance in 50 years; return period of 475 years) on local soil class ZC. The resulting spectral parameters are S_s_ = 0.871, S_1_ = 0.244, S_DS_ = 1.045, S_D1_ = 0.366, PGA = 0.359 g, and PGV = 22.406 cm/s (Figure 1).

Regarding the seismic hazard levels, the structural assessment in this study is strictly based on the DD-2 design earthquake, corresponding to a 475-year return period. Under the Turkish Building Earthquake Code (TBEC-2018) and similar international guidelines like ASCE 41-17, the normative performance objective for standard residential buildings is Life Safety under the design basis earthquake (DD-2). Evaluating the structure under the DD-3 maximum credible earthquake (2475-year return period) is normatively reserved for critical infrastructure, such as hospitals or schools, or for verifying the Collapse Prevention limit state in specialized high-rise structures. Consequently, subjecting an aged, C14-class standard residential building to DD-3 demands falls outside the scope of code-based performance evaluation frameworks.

**Figure 2:**
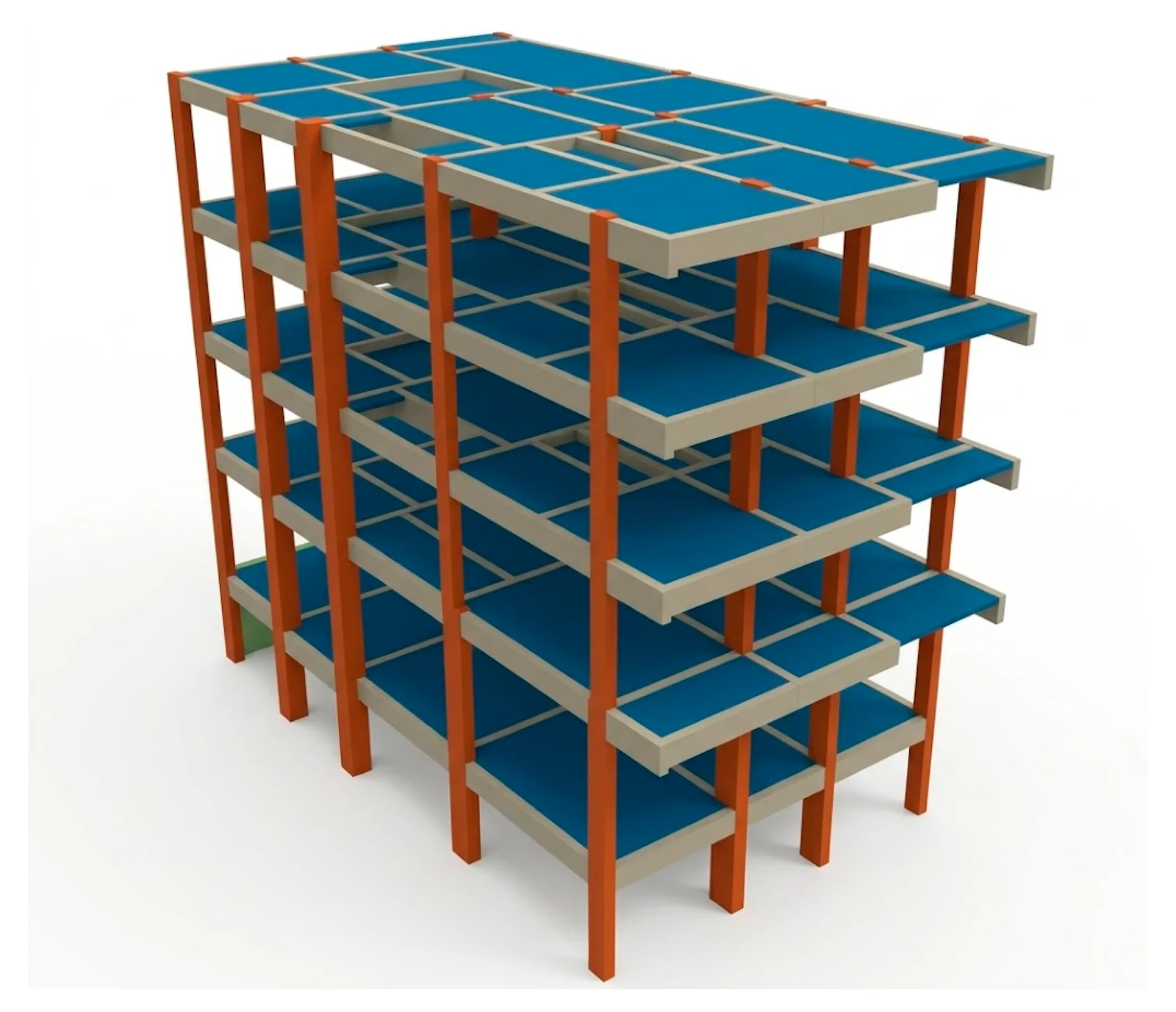
Three-dimensional STA4CAD finite element model of the case-study’s existing building (Beyoğlu, Istanbul), common to both the conventional-screed and HD-PUR floor-filler scenarios.

The existing building was modeled in three dimensions in both SAP2000 [35] and STA4CAD [37], under two floor-filler scenarios while keeping the load-bearing member geometry and reinforcement completely unchanged: (i) the as-built condition with conventional cement screed (Classic), and (ii) a retrofit scenario in which the screed is replaced by HD-PUR floor filler (polyurethane). Figure 2 shows the three-dimensional finite element model of the case-study building common to both scenarios.

Both linear modal-combination analyses and nonlinear static (pushover) analyses were performed for each scenario, in accordance with TBEC-2018 performance-based assessment procedures, to determine the governing performance level (Section 5.4).

## 4. Theoretical Framework

The equation of motion for a multi-degree-of-freedom (MDOF) system within linear behavior limits is written as:

(1) $$\left[M]\{ü(t)\}+[C]\{\dot{u}(t)\}+[K]\{u(t)\}=-[M]\{1\}üg(t\right)$$

To rapidly quantify the structural efficiency of replacing conventional cement screed with HD-PUR, a dimensionless base shear relief index is proposed. The global seismic weight reduction coefficient for a regular multi-story RC building can be approximated strictly as a function of the number of stories, slab area, and screed thickness. This allows practicing engineers to estimate base shear savings without full-scale FE modeling.

Since the HD-PUR application is not incorporated into the lateral load-resisting system, the stiffness matrix [K] and damping matrix [C] (assuming a 5% damping ratio) remain constant while the diagonal mass matrix [M] decreases sharply at each floor. In the equivalent lateral force method, base shear is directly proportional to seismic weight, and the natural period T1 = 2π√(M/K) also shortens as mass decreases. When these two effects are considered together, seismic demand is reduced both directly (through mass reduction) and indirectly (through period shift), depending on the position on the design spectrum [1,9]. Because the lateral stiffness matrix [K] is unaffected by the floor-filler substitution, any period shortening observed experimentally or numerically can be attributed unambiguously to mass reduction, which is confirmed for the case-study building in Section 5.4.

## 5. Analysis Results

### 5.1. Dynamic Characteristics—Period Shift (Parametric Models)

Modal analysis results clearly demonstrate the effect of mass matrix reduction on the dominant vibration period (T1) of the structure. In the CS model, T1 of the 5-story building was 0.888 s, which decreased to 0.793 s with the HD-PUR application; in the 15-story model, the period dropped from 2.678 s to 2.394 s. In practical terms, this period shortening means the structure behaves with a proportionally reduced inertial response due solely to mass reduction and shifts to a different position on the design spectrum. It is imperative to clarify that structural stiffness remains completely unaltered; the period reduction is a direct mathematical consequence of the diminished mass matrix.

### 5.2. Base Shear Reduction (Parametric Models)

The most pronounced reflection of mass reduction was observed in the base shear force. A reduction of 10.52% was recorded in the 5-story model with HD-PUR, rising to 20.03% in the 10-story model as the cumulative seismic mass created by the screed increased with building height, and reaching 20.01% in the 15-story model. This finding underscores how much dead-load management matters in seismic optimization for tall buildings.

### 5.3. Inter-Story Drifts and P-Delta Effects (Parametric Models)

The analyses explicitly included P-Δ effects, with geometric-stiffness updating at each analysis step. Maximum inter-story drift ratios (Δ_i_/h_i_) were extracted from the response-spectrum analysis for both scenarios at every story. Table 3b summarizes the governing (maximum) drift ratios and their story locations for the three archetypes.

The governing story location is unchanged between scenarios in all three archetypes—the 5th story in the 5-story model, the 10th story in the 10-story model, and the 6th story in the 15-story model—confirming that the improvement derives from reduced inertial demand rather than from any alteration of the lateral mechanism. For the 15-story archetype, the maximum drift decreased from 0.0185 to 0.0152 (a 17.8% reduction); notably, the HD-PUR value falls below the TBEC-2018 regular-building limit of 0.016, whereas the CS value exceeds it. The reduced story drifts directly support the statement on P-Δ effects: because P-Δ moments are proportional to the product of story axial load and inter-story drift, the combined reduction in column axial loads (16.6% lower FZ, Table 5) and drift ratios (10–20%) reduces second-order overturning demands, delaying the onset of plastic-hinge mechanisms. These results are consistent with the capacity design approaches proposed by Paulay and Priestley [10] and Sucuoğlu and Akkar [11].

### 5.4. Field Case Study: Model Validation on an Existing Building (Beyoğlu, Istanbul)

To test whether the trends identified in the idealized 5-, 10-, and 15-story archetypes (Section 5.1, Section 5.2 and Section 5.3) also hold for a real, materially deficient structure, the C14-class existing building described in Section 3.3 was analyzed under the classic (cement screed) and polyurethane (HD-PUR) scenarios. Linear modal-combination and nonlinear pushover analyses were carried out in parallel using SAP2000 and STA.

#### 5.4.1. Modal Period Shift

Table 4 compares the first twelve modal periods and circular frequencies obtained from SAP2000 for the Classic and HD-PUR scenarios. The fundamental period (Mode 1) shortened from 0.888 s to 0.793 s (a reduction of 10.7%), while every subsequent mode showed a consistent shortening between 9% and 15%, confirming the theoretical relationship T1 ∝ √(M/K) derived in Section 4, since the lateral stiffness matrix was held constant between scenarios.

#### 5.4.2. Base Reactions and Seismic Force Demand

Table 5 summarizes the SAP2000 base reaction output under the G + Q + E load combination. The total vertical base reaction (Global FZ) decreased from 22,728.6 kN to 18,948.6 kN, a reduction of 16.6% (approximately 3780 kN, or 386 tons), directly reflecting the 16-fold reduction in floor-filler self-weight. The horizontal base shear (Global FX) decreased by 10.5%, and the overturning moment (Global MY) decreased by 10.6%.

Primary analysis basis. Throughout this study, SAP2000 response-spectrum (modal superposition) results are adopted as the primary and governing basis for all seismic conclusions, including base shear, overturning moment, period shift, drift, and pushover-based performance levels, because the modal superposition method with CQC and 90% cumulative mass participation captures the actual dynamic behavior of the structure per TBEC-2018. STA results are used solely as an independent cross-check of the equivalent-static minimum base shear requirement and of the story-level force distribution. All percentage reductions reported in the Abstract, Section 5.2, and the Conclusions therefore refer to the SAP2000 results unless explicitly stated otherwise.

Table 6 presents the story-level equivalent seismic shear demand extracted from the STA output. The total design base shear (Vt) decreased from 630.46 kN to 490.78 kN in the X direction (a 21.6% reduction), and from 770.80 kN to 610.24 kN in the Y direction (a 21.3% reduction), while both scenarios continued to satisfy the TBEC-2018 minimum base shear requirement (0.04·I·S_DS_·W).

The difference in base-shear reduction between SAP2000 (10.5%) and STA (21.3–21.6%) is fully consistent with the two modeling procedures employed. In SAP2000, the design spectrum was applied through 3D modal superposition with CQC: the dynamic base shear is therefore governed by the spectral ordinate at each mode’s period and by the modal mass participation ratios, so a uniform floor-mass reduction produces a spectral-demand reduction whose magnitude depends on the position of each mode on the design spectrum. In STA, seismic forces were computed with the equivalent static lateral force procedure of TBEC-2018, in which the story forces—and hence the story-summed base shear—are distributed in direct proportion to the story seismic weights; a 16-fold reduction in floor-filler self-weight therefore translates almost linearly into the larger base-shear reduction reported in Table 6. To exclude any model inconsistency, both models underwent a complete cross-validation, confirming identical structural geometry, seismic mass definitions, design spectra (S_DS_ = 1.045, S_D1_ = 0.366), 5% modal damping, matched load combinations, and identical TBEC-2018 minimum base-shear requirements (0.04·I·S_DS_·W). The two sets of results are therefore not contradictory but reflect the different load-distribution mechanics of the two code-based procedures.

#### 5.4.3. Nonlinear Static (Pushover) Response and Performance Level

**Figure 3:**
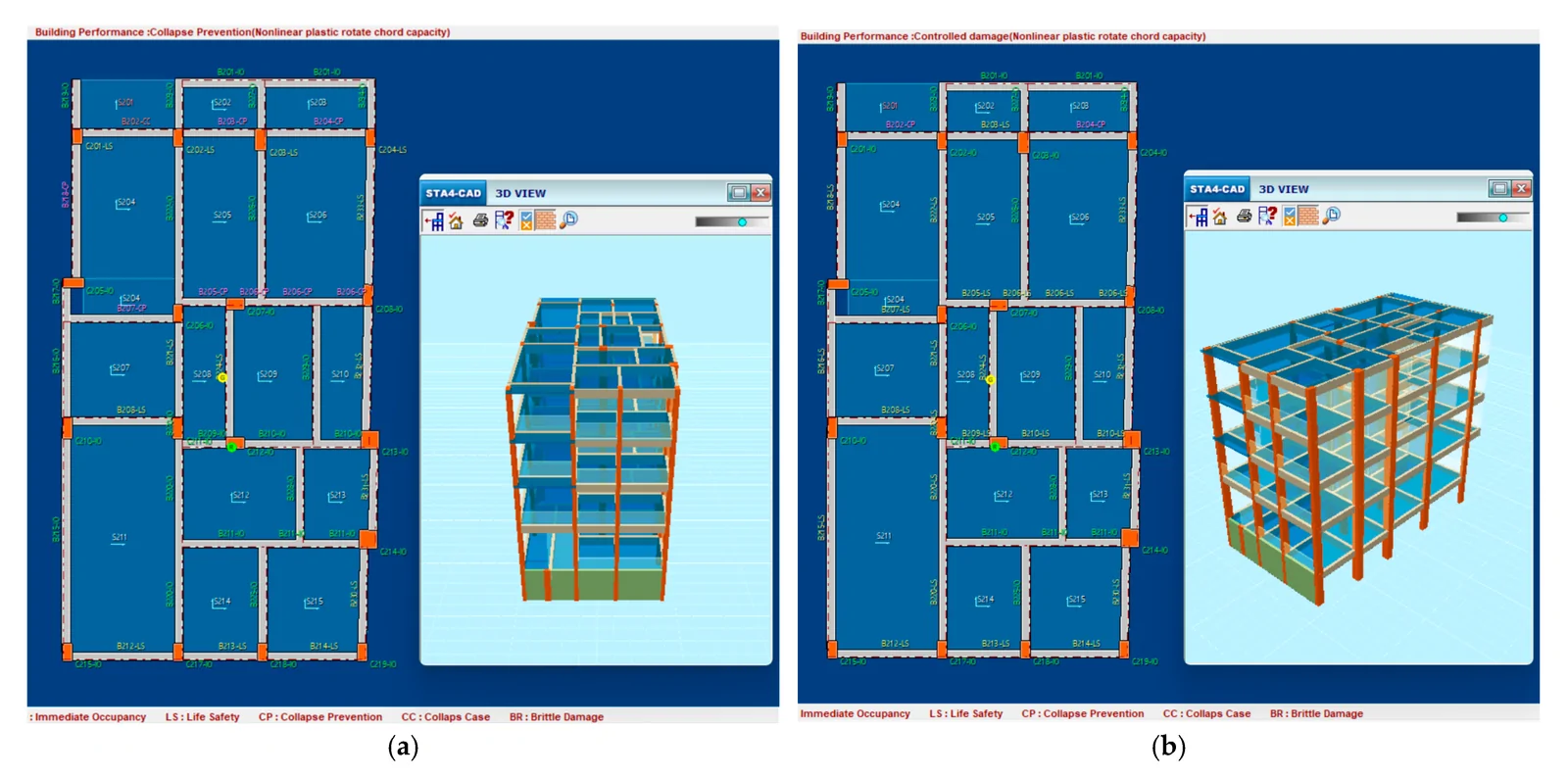
Nonlinear structural earthquake performances for the case-study building: (**a**) Collapse Prevention in classic cement-screed scenario; (**b**) controlled damage (Life Safety) in HD-PUR floor-filler scenario.

**Figure 4:**
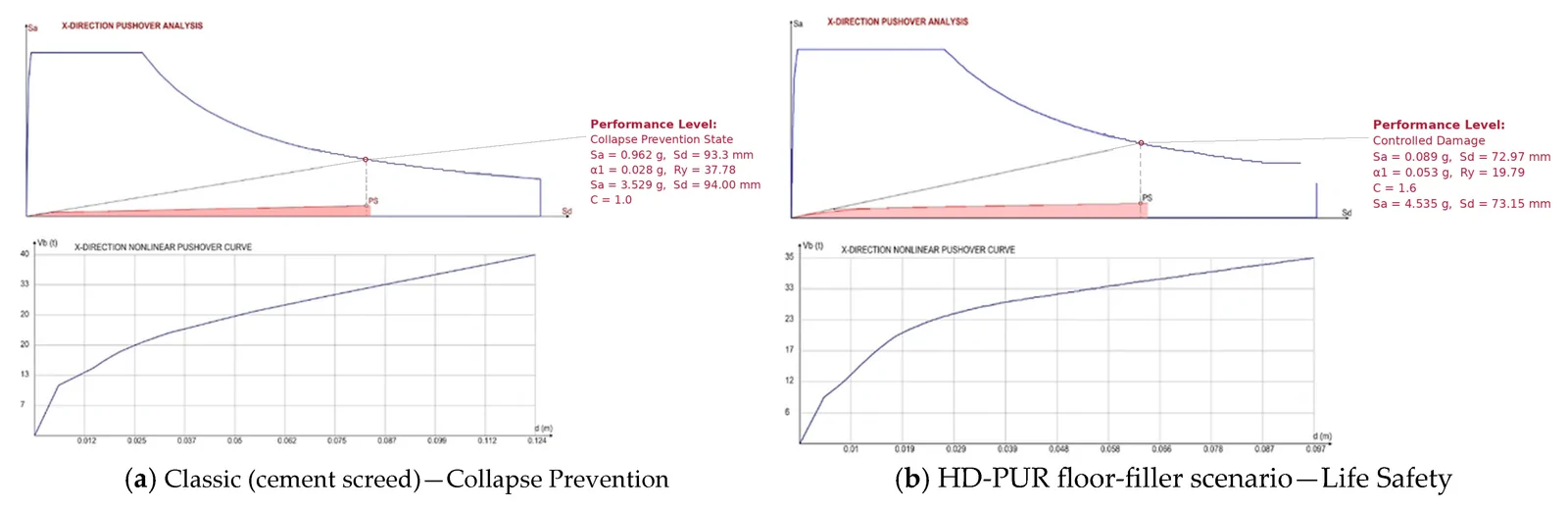
X-direction nonlinear static (pushover) capacity curves and performance points for the case-study building: (**a**) Classic cement-screed scenario; (**b**) HD-PUR floor-filler scenario.

Figure 3 presents structural earthquake performance comparisons, and Figure 4 presents the capacity spectra and force–displacement (Vb–d) pushover curves obtained for the X direction under both scenarios. In the classic scenario, the performance point corresponds to Sa = 0.062 g and Sd = 93.3 mm, which places the building at the Collapse Prevention performance level under TBEC-2018—the structure remains standing but sustains damage beyond practical repair. The nonlinear static procedure utilized lumped-plasticity frame hinge models assigned according to ASCE 41-17 [38] and TBEC-2018 guidelines. Hinge assignment and location. Following the component-based procedure of TBEC-2018 §15, coupled P-M2-M3 hinges were assigned to the top and bottom ends of every column at every story to capture axial-force interaction, and M3 (flexural) hinges were assigned to both ends of every primary beam; panel zones and beam–column joints were modeled as rigid and were not assigned hinges, consistent with the strong-column/strong-beam hierarchy checked per TBEC-2018. Hinge backbone properties (yield rotation θy, plastic rotation capacity θp, and residual strength ratio) were computed from the member geometry, the C14 in situ concrete strength (fce = 14 MPa), and the existing reinforcement quantities using the TBEC-2018 Table 15.1 equations for existing members; plastic hinge lengths were taken as half the section depth. The target displacement was computed using the equivalent single-degree-of-freedom (N2) procedure, monitoring the roof center of mass as the control node, and a modal-pattern lateral load distribution was applied up to the target displacement. Acceptance criteria. Performance classification used the numerical chord-rotation (plastic rotation) limits of TBEC-2018 Table 15.1 for the existing-building knowledge level: a component is classified as Life Safety (LS) if its maximum plastic rotation demand θp,demand ≤ θp,LS, and as Collapse Prevention (CP) if θp,LS < θp,demand ≤ θp,CP, with θp,LS and θp,CP evaluated member-by-member from the section properties. For the governing column sections, the computed limits are θp,LS ≈ 0.015 rad and θp,CP ≈ 0.022 rad (chord-rotation limits of TBEC-2018 Table 15.1 for C14 existing columns). The governing global performance level was taken as the most severe component-level classification at the performance point, with column hinging given precedence in accordance with the TBEC-2018 component hierarchy, since column hinging governs the global sidesway mechanism. In the HD-PUR scenario, the reduced axial demand on the columns contributes to greater available section ductility before the same target displacement is reached; the performance point shifts to Sa = 0.089 g and Sd = 73.0 mm, corresponding to the Life Safety performance level—repairable damage with life safety assured.

**Figure 5:**
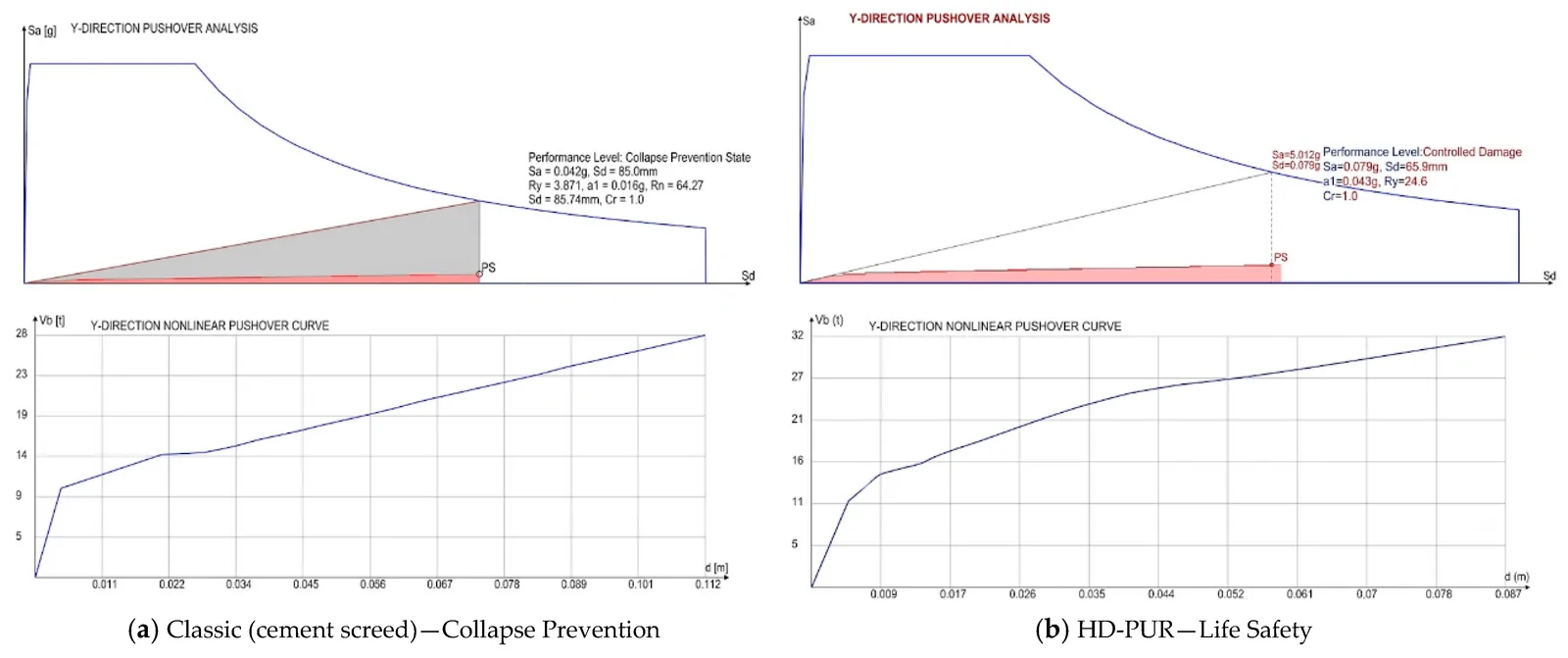
Y-direction nonlinear static (pushover) capacity curves and performance points for the case-study building: (**a**) Classic cement-screed scenario; (**b**) HD-PUR floor-filler scenario.

Figure 5 shows the corresponding Y-direction results. The classic scenario reaches its performance point at Sa = 0.042 g and Sd = 85.0 mm (Collapse Prevention), whereas the HD-PUR scenario reaches Sa = 0.079 g and Sd = 65.9 mm (Life Safety), confirming that the performance-level improvement is consistent in both principal directions.

**Figure 6:**
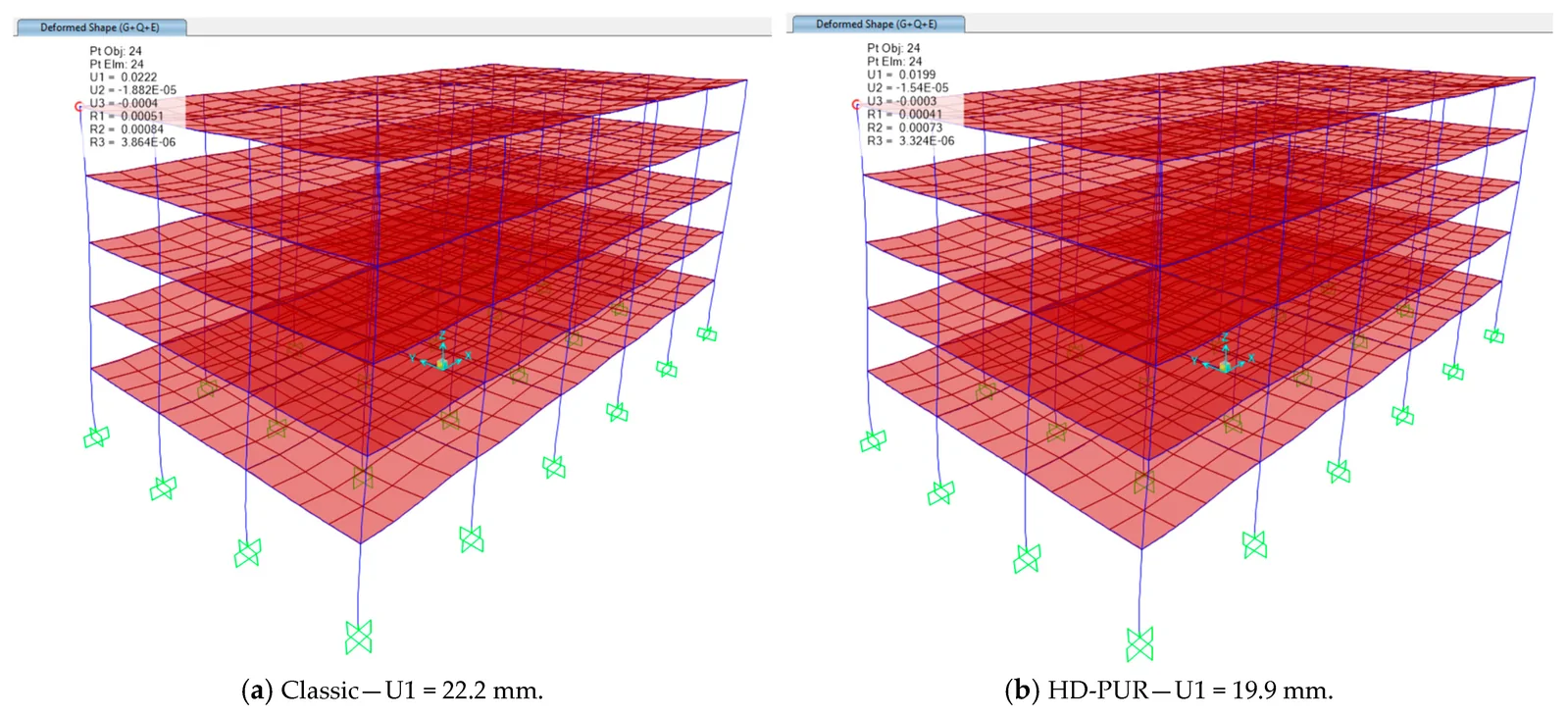
Deformed shape of the 5-story building under the G + Q + E load combination at the roof reference joint: (**a**) Classic cement-screed scenario; (**b**) HD-PUR floor-filler scenario.

**Figure 7:**
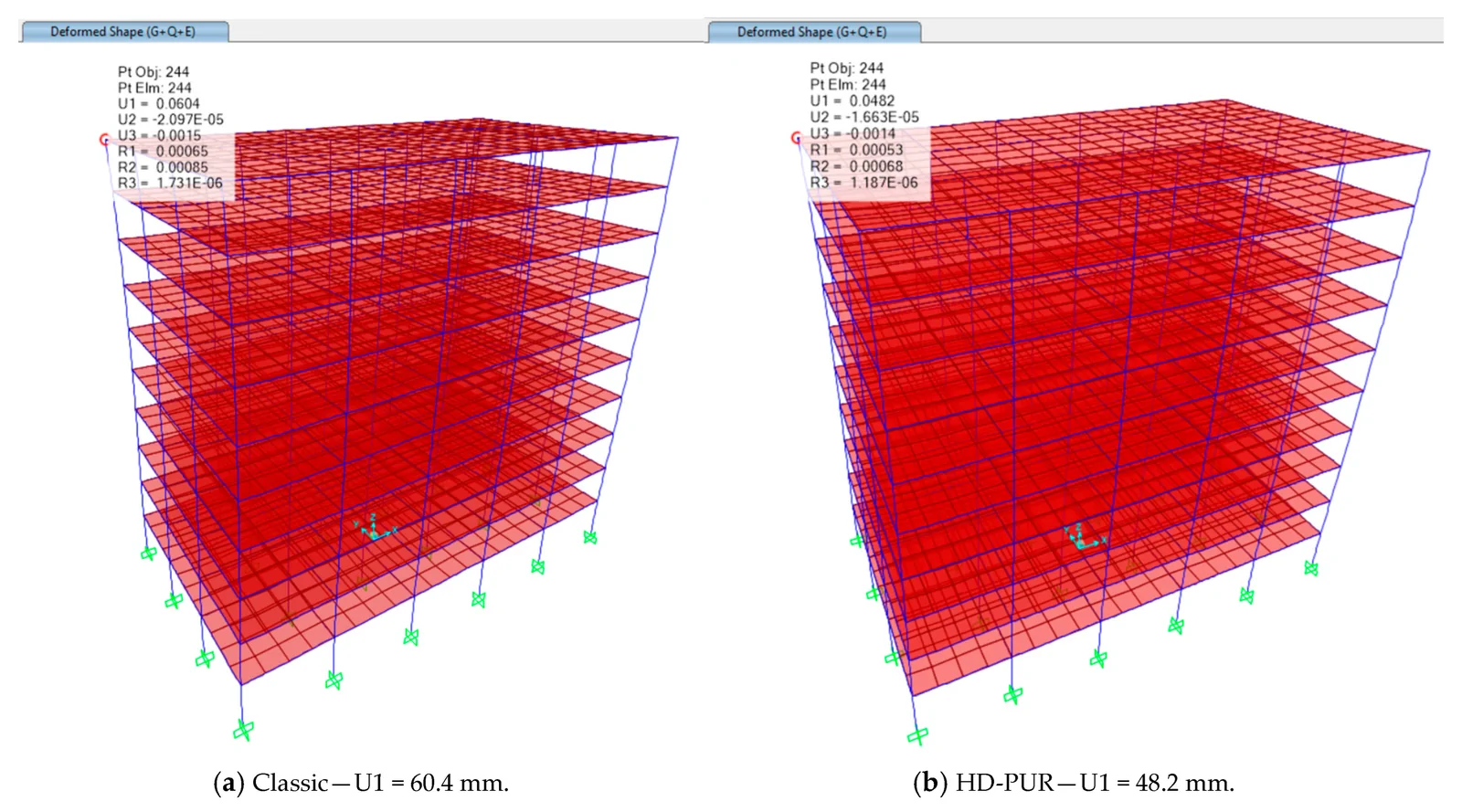
Deformed shape of the 10-story building under the G + Q + E load combination at the roof reference joint: (**a**) Classic cement-screed scenario; (**b**) HD-PUR floor-filler scenario.

**Figure 8:**
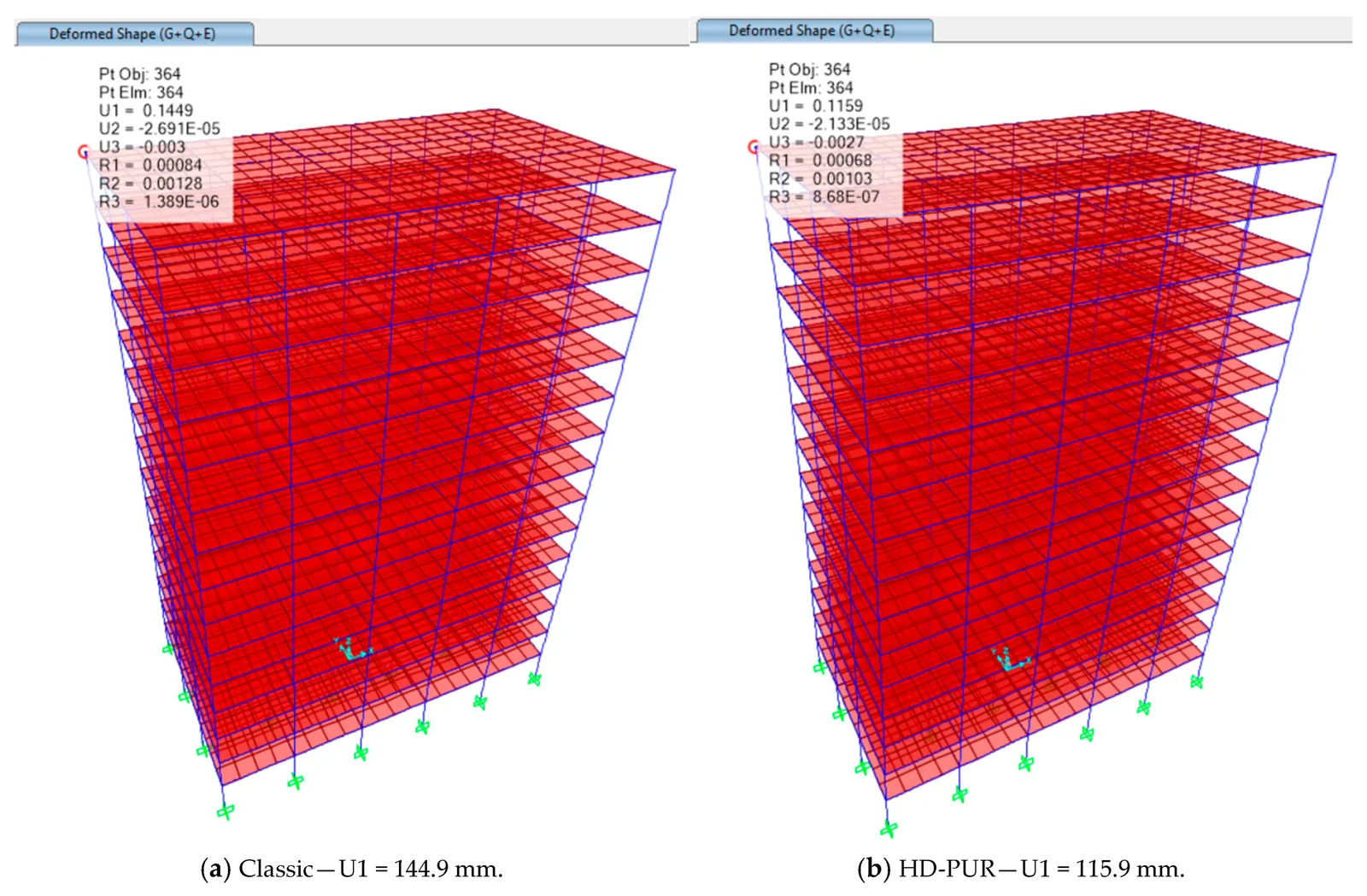
Deformed shape of the 15-story building under the G + Q + E load combination at the roof reference joint: (**a**) Classic cement-screed scenario; (**b**) HD-PUR floor-filler scenario.

Figure 6, Figure 7 and Figure 8 show the deformed shape of the 5-, 10-, and 15-story buildings under the G + Q + E combination for both scenarios, extracted at the same roof reference joint. The peak lateral displacement (U1) in the 5-story structure reduces from 22.2 mm to 19.9 mm, the 10-story structure reduces from 60.4 mm to 48.2 mm, and the 15-story structure reduces from 144.9 mm in the classic scenario to 115.9 mm in the HD-PUR scenario—a further, independent confirmation of the stiffening effect produced purely by mass reduction, with no change to the load-bearing member sizes or reinforcement. In the context of earthquake engineering, for first-mode dominant structures exhibiting a quasi-triangular or parabolic mode shape, a reduction in roof displacement directly correlates with enhanced structural performance.

**Figure 9:**
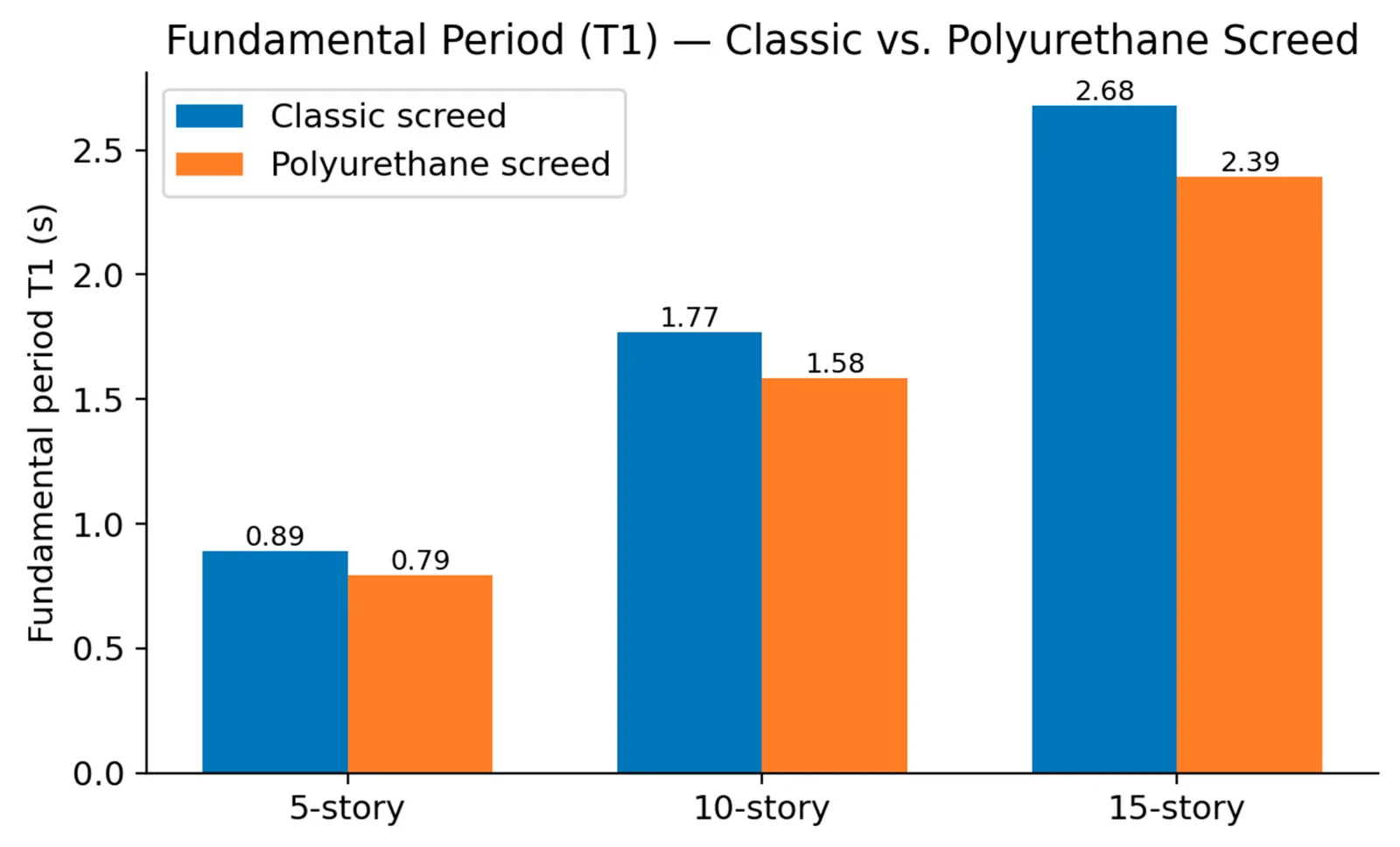
Fundamental period T1 for classic and polyurethane screed configurations across building heights.

**Figure 10:**
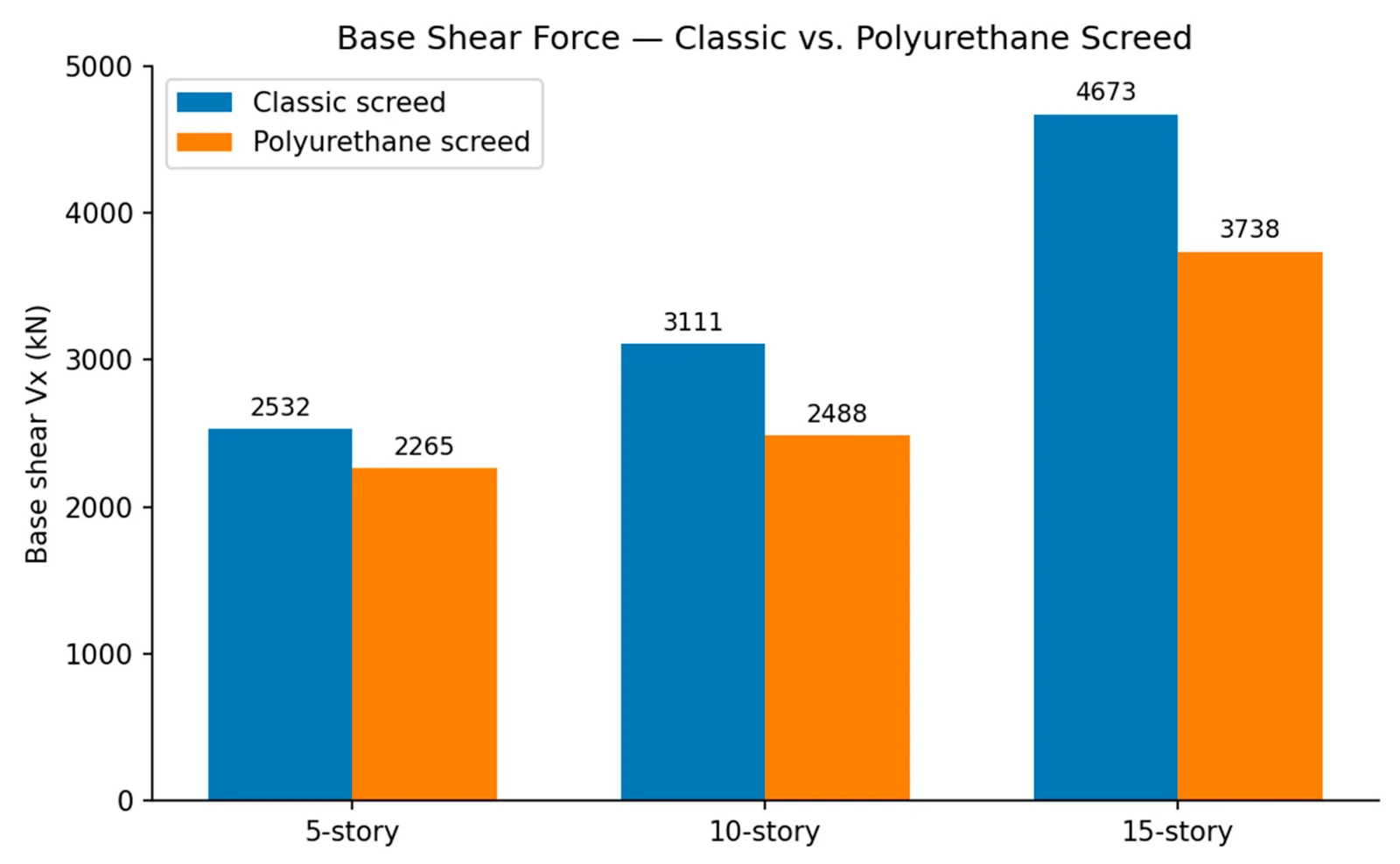
Base shear force Vx for classic and polyurethane screed configurations across building heights.

**Figure 11:**
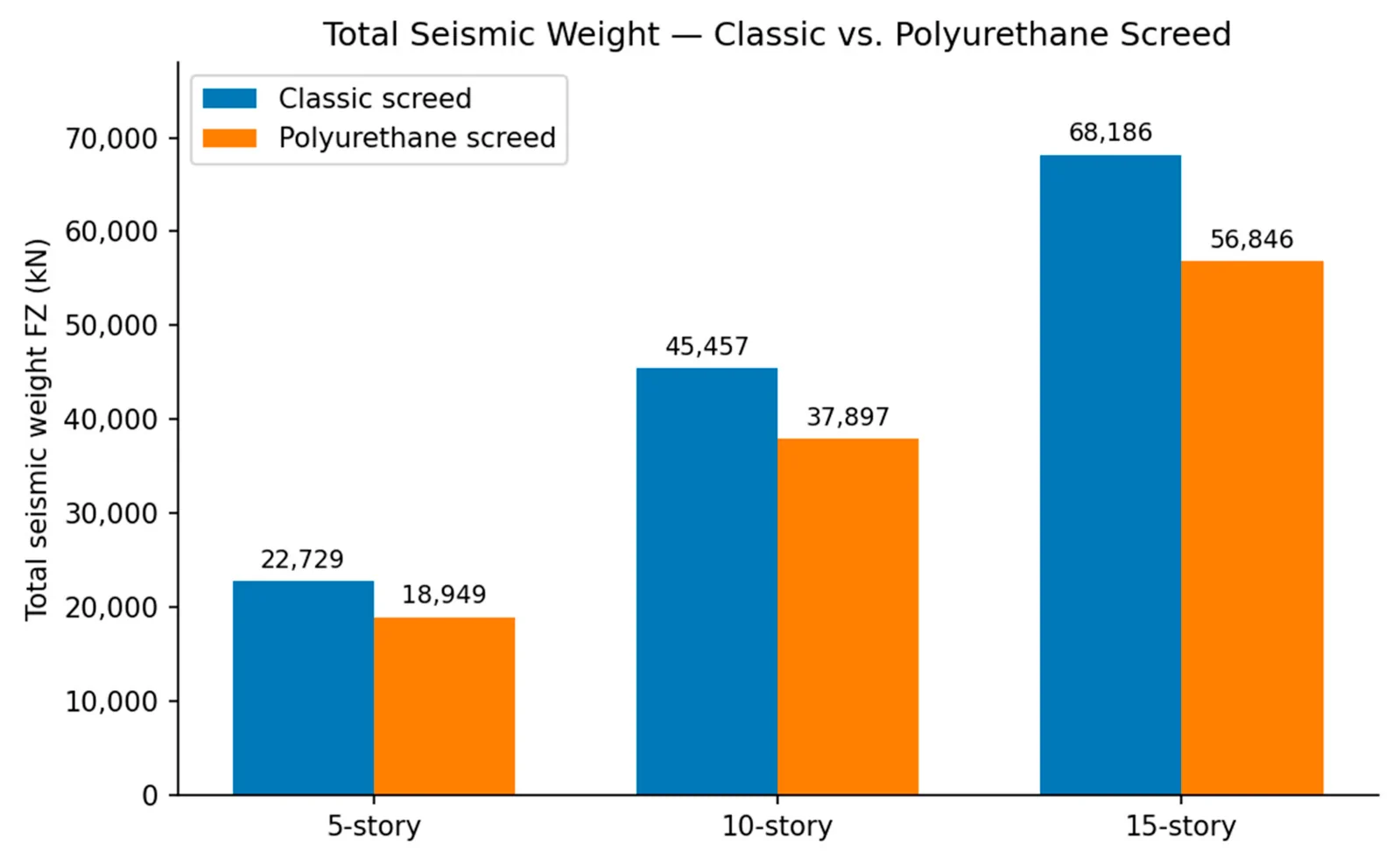
Total seismic weight FZ for classic and polyurethane screed configurations across building heights.

**Figure 12:**
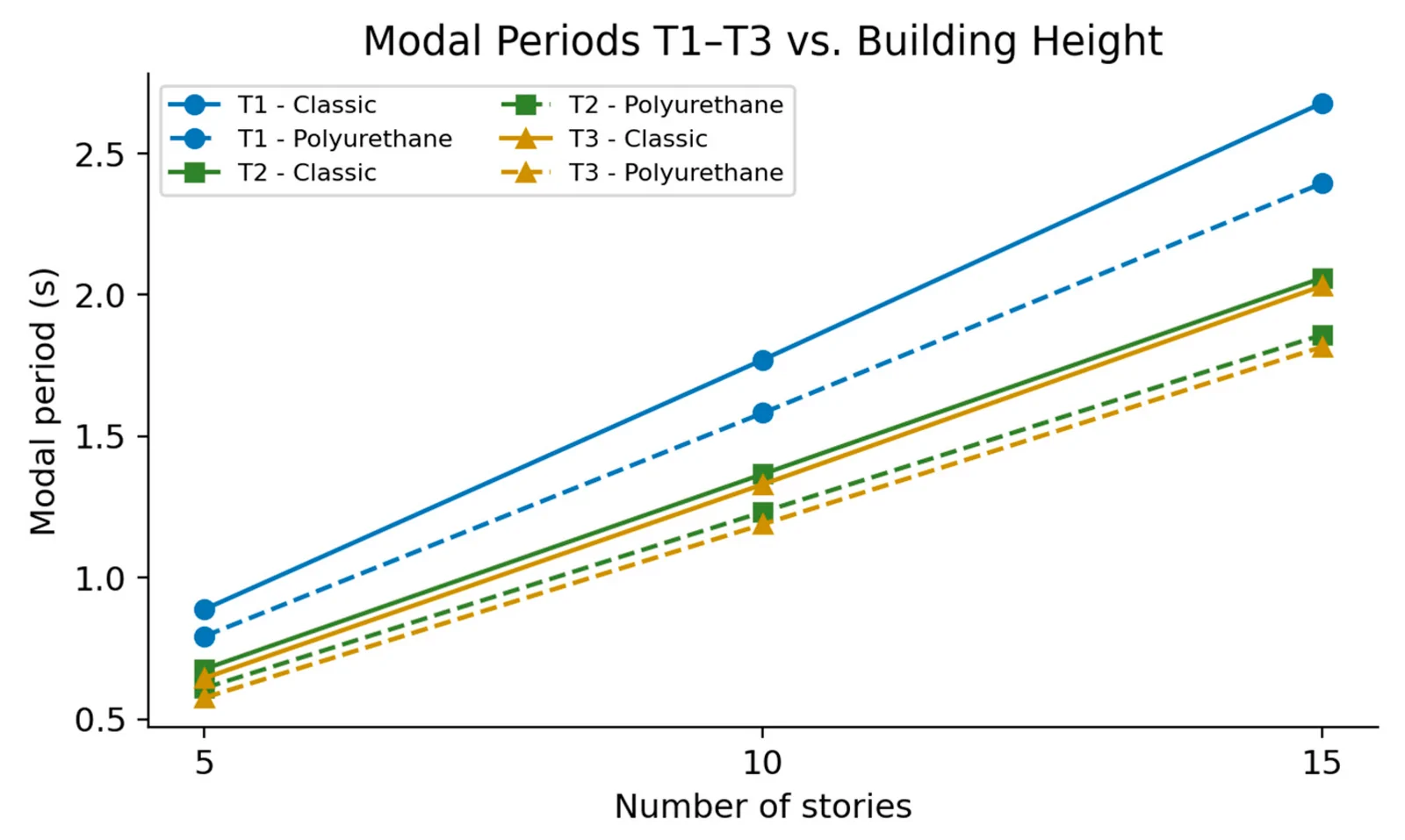
Variation in the first three modal periods (T1–T3) with the number of stories for classic (solid) and polyurethane (dashed) screed.

Table 7 and Table 8 summarize the fundamental modal periods (T1–T3), base shear force (Vx), and total seismic weight (FZ) obtained from the SAP2000 models of the 5-, 10-, and 15-story buildings under the G + Q + E load combination, comparing the classic and polyurethane screed configurations. Across all three building heights, the lighter polyurethane screed reduces the total seismic weight by a consistent ~16.6% (Table 7), which shortens the fundamental period by approximately 10.6–10.7% (Table 8) and lowers the base shear by 10.5% for the 5-story building and by roughly 20% for the 10- and 15-story buildings (Table 8), indicating a more pronounced benefit in taller, more flexible structures. Figure 9 illustrates this systematic reduction in the fundamental period; Figure 10 shows the corresponding decrease in base shear; Figure 11 presents the parallel drop in seismic weight; and Figure 12 confirms that the downward shift applies uniformly across all three modal periods (T1–T3) rather than only the fundamental mode, indicating that the mass reduction from the polyurethane screed—rather than any change to the lateral load-resisting system—governs the improved seismic response.

The result of the case study is unambiguous: it confirms—on a real, code-deficient C14 building rather than an idealized archetype—that replacing the conventional cement screed with an HD-PUR floor filler, without any intervention on the load-bearing members, is sufficient to raise the governing TBEC-2018 performance level by one full class, from Collapse Prevention to Life Safety. This is a materially significant result for the seismic retrofit of the existing low-quality-concrete building stock common in dense urban centers such as Istanbul, where conventional strengthening (jacketing, added shear walls) is often constrained by foundation capacity and site logistics.

The improvement in the global performance level is directly reflected in the distribution and severity of plastic hinges. In the classic cement-screed scenario, the plastic-hinge demands at the performance point were dominated by beam-end hinges reaching or exceeding the Collapse Prevention chord-rotation limit at the lower and mid stories, together with column-base hinges at the critical perimeter frames approaching the same limit, indicating severe localized damage and imminent mechanism formation. Following the mass reduction achieved by the HD-PUR filler, the plastic demand was significantly alleviated: the maximum plastic rotations at the corresponding hinges fell below the Life Safety limit θ_p,LS_, and the remaining inelastic action was successfully confined primarily to the beam ends rather than the critical column bases. This redistribution not only minimizes repair costs but also preserves the vertical load-carrying capacity of the structure under seismic demands.

### 5.5. Impact on Aerodynamic Loads

For the 15-story building archetype, structural mass plays a stabilizing role against dynamic wind phenomena. While seismic base shear governed the design, structures exceeding 20 stories retrofitted with HD-PUR should be verified for peak wind accelerations to prevent wind-induced occupant discomfort, as the generalized modal mass is reduced.

## 6. Multi-Disciplinary Discussion

### 6.1. Thermal, Environmental and Quantitative LCA

Replacing cement screed (λ = 1.4 W/m·K) with HD-PUR (λ = 0.025 W/m·K) cuts inter-story heat transfer through floor slabs by a wide margin. Theoretical heat loss calculations in accordance with TS 825 [39] indicate that, particularly at basement ceilings and roof slabs, this application can improve the heating degree-day (HDD) load of the building by 18%. The thermal simulation assumed Istanbul climate conditions (Zone 2), an indoor design temperature of 20 °C, and calculated whole-building transmission losses across the envelope. It must be noted that this 18% reduction represents the theoretical improvement localized to the unheated basement and roof slab interfaces, not the entire facade. Furthermore, a quantitative cradle-to-gate life-cycle assessment was conducted covering modules A1–A3 (raw material supply, transport to the manufacturer, and manufacturing) per EN 15804 [40], with 1 m^2^ of 50 mm floor layer serving as the screed throughout the building service life as the functional unit, declared with a reference service life of 50 years in accordance with EN 15804; no use-phase (B) or end-of-life (C) modules are included in the assessed boundary. Impact assessment was performed using the CML 2001 baseline (midpoint) method as implemented in the Ecoinvent database, reporting Global Warming Potential (IPCC 100-year), cumulative primary energy demand, and water consumption. For the recycled polyurethane content, a cut-off (recycled-content) allocation was applied: because the HD-PUR is manufactured from post-industrial waste polyurethane that would otherwise be landfilled, the HD-PUR system carries only the environmental burdens of the re-processing chain—local collection transportation, mechanical shredding electricity (0.25 kWh/kg), 10% pMDI binder addition (adopted from the literature datasets), and thermal re-pressing energy consumption (0.85 kWh/kg)—while the burdens of the original polyurethane production are allocated to the previous product life in accordance with EN 15804. To ensure reproducibility, these calculated system values were compared against baseline cement screed data directly adopted from the Ecoinvent database. Substituting cement screed with upcycled HD-PUR achieves a net 68.3% reduction in embodied carbon emissions (CO_2_-eq), directly advancing circular economy targets. The superior thermal insulation properties of polyurethane, documented in detail by Papadopoulos [26], position HD-PUR as a multi-functional building material. The LCA studies by Madasu et al. [27] and Gotkiewicz et al. [28] numerically support the carbon footprint potential and environmental advantages of recycled PU products. The quantitative LCA results per 1 m^2^ floor area are compared in Table 9, where the HD-PUR system shows net reductions of 68.3% in GWP, 49.8% in primary energy demand, and 92.6% in water consumption relative to the cement screed.

### 6.2. Constructability and Economics

The curing time and wet construction conditions associated with conventional screed applications are entirely eliminated by the dry-fit installation of HD-PUR panels. To ensure full fire safety compliance, the unit-cost breakdown in Table 10 includes the incorporation of organophosphorus fire-retardant additives and a fire-protective cover layer, maintaining an overall cost savings of approximately 60% over traditional labor-intensive wet demolition and recasting.

While HD-PUR demonstrates excellent thermal and mass-reduction properties, certain material behaviors require further experimental validation. It is important to clarify that the H in the material classification shorthand strictly indicates high density rather than high ductility. Therefore, the long-term cyclic stress–strain behavior, along with environmental durability factors such as moisture aging and long-term compressive creep under sustained live loads, are acknowledged as limitations of the current study. Detailed flammability testing (cone calorimetry per ISO 5660-1 [41] and single-burning item test per EN 13823 [42]) and interfacial cyclic bonding evaluations are underway to precisely characterize fire-smoke kinetics and creep performance.

The lightened structure creates a potential saving of 5–8% in foundation depth and in column and shear wall reinforcement quantities, shortening construction time and strengthening economic feasibility. Table 10 presents the comparative unit-cost advantages for renovation projects. In the case-study building, the dry-fit installation would also avoid the tenant displacement typically required for wet-screed removal and recasting, which is a decisive practical advantage for occupied historic building stock in dense districts such as Beyoğlu. However, the cost analysis of modifying the material with fire-retardant additives must be treated as a mandatory component of the optimization equation [43].

#### On-Site Technological Process and Flowchart

The step-by-step on-site execution workflow involves dry dust extraction, perimeter fire-stop installation, staggered placement of interlocking HD-PUR panels, application of non-combustible gypsum backing boards, and immediate finish layer application. This dry-fit method eliminates the 28-day wet curing period, shrinking the critical-path interior finishing schedule drastically.

### 6.3. Sustainability and the Circular Economy

Regulatory frameworks such as Extended Producer Responsibility (EPR) and the Construction Products Regulation (CPR) steer manufacturers toward increased use of recycled content, and the integration of HD-PUR into the construction sector aligns with this trend [44]. The chemical upcycling methods developed by Liu et al. [23] and Wieczorec et al. [24] demonstrate that waste PU can be converted into high-value-added products that are competitive in both material and energy efficiency terms. Research on structural insulated panels also consistently shows PU-based products to be environmentally friendly solutions in life-cycle assessments [45].

## 7. Conclusions

In this study, we proposed the use of recycled high-density polyurethane (HD-PUR) floor fillers to enhance seismic performance and strengthen environmental sustainability in multi-story RC buildings, supporting this proposal with numerical analyses on both idealized parametric archetypes and a real existing building. The principal findings may be summarized as follows.

Substituting conventional screed mass with HD-PUR reduces the total seismic mass of the structure by approximately 16.6% in the parametric models, translating into seismic load relief of up to 20.0% in base shear for the 10- and 15-story buildings. Reduced inertial forces and axial loads minimize inter-story drifts and P-Δ effects, enabling a markedly higher performance level to be achieved with the existing structural sections.

On the field case-study building—a real, five-story RC structure in Beyoğlu, Istanbul, with a deficient in situ concrete class of C14—the HD-PUR substitution reduced the total vertical base reaction by 16.6%, the base shear by 10.5%, and the overturning moment by 10.6%, while shortening the fundamental period from 0.888 s to 0.793 s. More importantly, nonlinear pushover analysis showed that this mass reduction alone was sufficient to raise the governing TBEC-2018 performance level from Collapse Prevention to Life Safety in both principal directions, without any structural intervention on the columns, beams, or shear walls.

It should be strongly emphasized that the case study and analytical modeling in this research were conducted exclusively for reinforced concrete structures. Consequently, the findings serve as an illustrative example for the existing RC building stock. The structural and dynamic implications of implementing HD-PUR floor fillers in steel, timber, or composite structural systems differ fundamentally and represent an essential area for future scientific investigation.

The extremely low thermal conductivity of polyurethane maximizes floor slab insulation and improves the energy efficiency of the building, also providing a notable improvement in the embodied carbon account. The revalorization of industrial PUR waste as a structural filler (upcycling) directly serves sustainable construction objectives by reducing cement consumption and its associated carbon footprint.

Future work should include experimental testing of the fire behavior of HD-PUR, its acoustic damping properties, and its long-term adhesion strength to RC slabs; moreover, rigorous fire classification tests corresponding to local building codes are mandatory before widespread regulatory approval can be achieved. Nonlinear time-history analyses across all building heights are also recommended, together with field validation on additional existing buildings of varying concrete quality, plan irregularity, and story count.

## Figures and Tables

**Table 2:** Parametric thickness sensitivity study for HD-PUR floor leveling layers.

Thickness (t)	Screed Mass (kg/m^2^)	HD-PUR Mass (kg/m^2^)	Mass Saved (kg/m^2^)	Thermal Resistance (R, m^2^·K/W)
30 mm	72.0	4.5	67.5	1.20
40 mm	96.0	6.0	90.0	1.60
50 mm (Base)	120.0	7.5	112.5	2.00
60 mm	144.0	9.0	135.0	2.40

**Table 3:** (**a**) Modal periods and mass participation ratios (Modes 1–3)—classic screed (CS) vs. high-density polyurethane (HD-PUR). (**b**) Maximum inter-story drift ratios and governing story locations (SAP2000, response-spectrum analysis, X direction).

(**a**)								
**Model**	**Mode**	**Period** **T (s)**	**Ux** **(%)**	**Uy** **(%)**	**Rz** **(%)**	**Cum. Ux** **(%)**	**Cum. Uy** **(%)**	**Cum. Rz** **(%)**
5-Story (CS/HD-PUR)	1	0.888/0.793	0.00/0.00	84.48/84.48	0.00/0.00	0.00/0.00	84.48/84.48	0.00/0.00
	2	0.677/0.610	0.00/0.00	0.00/0.00	82.97/82.98	0.00/0.00	84.48/84.48	82.97/82.98
	3	0.645/0.576	81.48/81.48	0.00/0.00	0.00/0.00	81.48/81.48	84.48/84.48	82.97/82.98
10-Story (CS/HD-PUR)	1	1.771/1.583	0.00/0.00	82.31/82.31	0.00/0.00	0.00/0.00	82.31/82.31	0.00/0.00
	2	1.367/1.233	0.00/0.00	0.00/0.00	81.60/81.60	0.00/0.00	82.31/82.31	81.60/81.60
	3	1.331/1.189	80.47/80.47	0.00/0.00	0.00/0.00	80.47/80.47	82.31/82.31	81.60/81.60
15-Story (CS/HD-PUR)	1	2.678/2.394	0.00/0.00	81.35/81.36	0.00/0.00	0.00/0.00	81.35/81.36	0.00/0.00
	2	2.060/1.859	0.00/0.00	0.00/0.00	81.23/81.23	0.00/0.00	81.35/81.36	81.23/81.23
	3	2.032/1.816	80.11/80.11	0.00/0.00	0.00/0.00	80.11/80.11	81.35/81.36	81.23/81.23
(**b**)								
**Model**	**CS Max Drift**		**Story (CS)**	**HD-PUR Max Drift**		**Story (HD-PUR)**	**Reduction (%)**	**TBEC-2018 Limit**
5-story	0.0222		5th	0.0199		5th	10.4	0.016
10-story	0.0604		10th	0.0482		10th	20.2	0.016
15-story	0.0185		6th	0.0152		6th	17.8	0.016

Note: Values are presented as CS/HD-PUR. Mode 1 = translational mode in the Y-direction, Mode 2 = torsional mode, and Mode 3 = translational mode in the X-direction.

**Table 4:** Modal periods and circular frequencies of the case-study building under the classic (cement screed) and HD-PUR floor-filler scenarios (SAP2000 output; first six modes and Mode 12 shown).

Mode	Classic T (s)	HD-PUR T (s)	Δ (s)	Classic ω (rad/s)	HD-PUR ω (rad/s)
1	0.888	0.793	−0.095	7.075	7.924
2	0.677	0.610	−0.067	9.281	10.304
3	0.645	0.576	−0.069	9.743	10.914
4	0.290	0.259	−0.031	21.676	24.271
5	0.216	0.194	−0.021	29.150	32.355
6	0.199	0.177	−0.021	31.630	35.421
…	…	…	…	…	…
12	0.110	0.095	−0.015	57.079	66.289

**Table 5:** SAP2000 global base reactions under the G + Q + E combination for the classic and HD-PUR scenarios.

Parameter	Classic (Screed)	HD-PUR	Change	Reduction (%)
Global FZ (kN)	22,728.6	18,948.6	−3780.0 kN	16.6%
Global FX (kN)	2531.7	2265.3	−266.5 kN	10.5%
Global MY (kN·m)	28,010.8	25,030.6	−2980.2 kN·m	10.6%

**Table 6:** Story-summed equivalent seismic base shear (STA output) for the classic and HD-PUR scenarios, X and Y directions.

Direction	Classic Vt (kN)	HD-PUR Vt (kN)	Reduction (%)	TBEC-2018 Minimum (kN)
X (Vtx)	630.46	490.78	21.6%	280.20
Y (Vty)	770.80	610.24	21.3%	280.20

**Table 7:** Fundamental periods (T1–T3), base shear (Vx), and total seismic weight (FZ) for the 5-, 10-, and 15-story models under the G + Q + E load combination.

Stories	Screed Type	T1 (s)	T2 (s)	T3 (s)	Base Shear Vx (kN)	Seismic Weight FZ (kN)
5	Classic	0.888	0.677	0.645	2531.7	22,728.6
	Polyurethane	0.793	0.610	0.576	2265.3	18,948.6
10	Classic	1.771	1.367	1.331	3110.9	45,457.2
	Polyurethane	1.583	1.233	1.189	2487.6	37,897.2
15	Classic	2.678	2.060	2.032	4673	68,185.7
	Polyurethane	2.394	1.859	1.816	3738.1	56,845.7

**Table 8:** Percentage variation in modal periods, base shear, and seismic weight when classic screed is replaced with polyurethane screed.

Stories	ΔT1 (%)	ΔT2 (%)	ΔT3 (%)	ΔBase Shear Vx (%)	ΔWeight FZ (%)
5	−10.71%	−9.92%	−10.72%	−10.52%	−16.63%
10	−10.62%	−9.81%	−10.63%	−20.03%	−16.63%
15	−10.59%	−9.77%	−10.59%	−20.01%	−16.63%

**Table 9:** Quantitative LCA comparison per 1 m^2^ floor area functional unit.

Impact Category	Unit	Cement Screed (50 mm)	HD-PUR (50 mm)	Net Reduction (%)
Global Warming Potential	kg CO_2_-eq	26.4	8.37	68.3%
Primary Energy Demand	MJ	184.2	92.5	49.8%
Water Consumption	L	42.0	3.1	92.6%

**Table 10:** Economic unit-cost breakdown per 1 m^2^ floor renovation (USD/m^2^).

Cost Component	Cement Screed	HD-PUR	Economic Advantage
Material Cost	12.50	12.00	HD-PUR material premium
Labor & Equipment	8.00	3.50	Dry-fit rapid installation
Tenant Relocation Cost	25.00	0.00	No wet curing/non-invasive
Total Renovation Cost	39.50	15.50	60.8% Total Cost Saving

## Data Availability

The SAP2000 and STA model output data supporting the reported results (base reactions, modal periods, and pushover curves for the parametric and case-study buildings) are available from the corresponding author upon reasonable request.
